# Supercritical Fluid Extraction (SFE) of Polar Compounds from *Camellia sinensis* Leaves: Use of Ethanol/Water as a Green Polarity Modifier

**DOI:** 10.3390/molecules28145485

**Published:** 2023-07-18

**Authors:** Sirine Atwi-Ghaddar, Lydie Zerwette, Emilie Destandau, Eric Lesellier

**Affiliations:** Institute of Organic and Analytical Chemistry (ICOA), University of Orléans, CNRS UMR7311, 45100 Orléans, France; sirine.atwi-ghaddar@univ-orleans.fr (S.A.-G.); lydie.zerwette@univ-orleans.fr (L.Z.); emilie.destandau@univ-orleans.fr (E.D.)

**Keywords:** green tea, caffeine, catechins, epigallocatechin gallate, water additive, natural ingredients, Box–Behnken design, polar modifiers, sequential selective extraction

## Abstract

The use of bioactive plant extracts in cosmetic products is a common practice. Most of these extracts are obtained by maceration in organic solvents, and depending on which solvents are used, the polarity and the structure of the target molecules will vary. Polyphenols are polar compounds that often display antioxidant and/or antibacterial activities. To extract them, ethanol/water mixtures are usually selected as green solvents. This solid–liquid extraction (assisted or not) requires the use of high volumes of solvents and many additional steps like mixing, agitation, filtration, and evaporation. Alternatively, supercritical carbon dioxide (SC-CO_2_) offers many benefits for plant extraction: economical, non-toxic, and naturally concentrated extracts. However, its low polarity is not suitable to solubilize polar compounds. In this study, an experimental design was used to optimize supercritical fluid extraction (SFE) of caffeine and catechins from *Camellia sinensis*. Catechins are recognized for skin care use (antioxidant) and caffeine is also used for its skin care properties and to prevent excess storage of fat in cells. The temperature, modifier content, and water additive percentage were used as independent variables. The results showed that while the temperature was an insignificant parameter, a higher percentage of water (up to 20% in ethanol) and modifier favored the extraction of the polar target molecules. Additionally, the SFE results were compared with ultrasound-assisted extraction (UAE). Finally, a sequential selective extraction of caffeine from catechins is also presented.

## 1. Introduction

Tea (*Camellia sinensis*) is largely consumed around the world. The beverage obtained by the infusion of leaves in hot water (80–95 °C) contains numerous polyphenols that have several biological activities, providing beneficial effects on human health [1,2,3,4,5].

**Figure 1:**
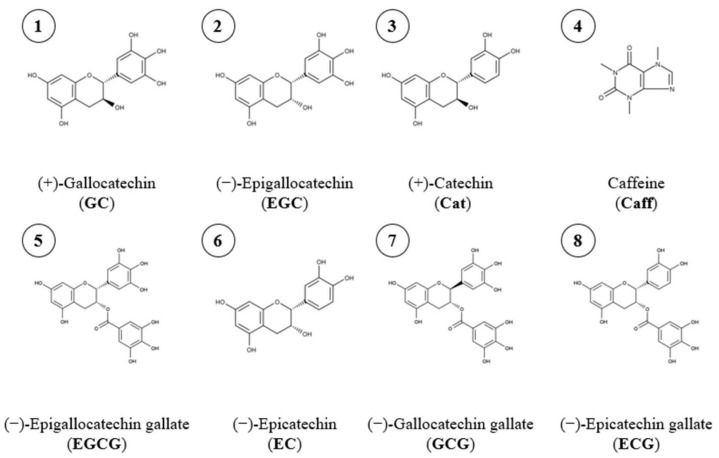
Chemical structure of green tea polar compounds.

The major bioactive compounds are catechins, which are flavan-3-ols, a type of secondary metabolite (Figure 1). The tea leaves are processed to make green tea, such as by roasting or steaming. Additional processes like fermentation (semi or fully) of leaves produce different variations of tea, for example, oolong tea and black tea, in which the content of catechins can decrease [3,4]. The structural differences for these catechins are the hydroxyl group number on the B benzene ring (catechol), two for catechin (Cat) and catechin gallate (CG), or three for gallocatechin (GC) and gallocatechin gallate (GCG), and the presence of esterified gallic acid of the hydroxyl group in position 3 on the C ring (dihydropyran heterocycle), leading to the two gallate forms, CG and GCG of Cat and GC. Moreover, due to the presence of two asymmetric carbons, number 2 and 3 on the C ring, these four catechins can be found in the *cis* form, called «epi», which is less stable than the *trans* form [1]. Furthermore, in green tea leaves, the four main flavanols are epicatechin (EC), epigallocatechin (EGC), epicatechin gallate (ECG), and epigallocatechin gallate (EGCG), with the two last being the most abundant [1,2,3,4,5].

The analysis of these compounds is generally achieved using high- or ultra-high-performance liquid chromatography (HPLC/UHPLC), with C18-bonded stationary phases and either water/acetonitrile [6,7,8,9,10,11,12,13], water/methanol [14,15,16,17], or water/ACN/MeOH [18] elution gradient. The retention order generally follows the same pattern, CG/ECG/Cat/EC/EGCG/GCG/ECG/CG, meaning that the free esterified forms (CG and Cat) elute before the esterified ones, GCG and CG, and for the two classes of catechins, the compounds with three hydroxyl groups on the B ring elute before the ones having two hydroxyl groups. However, when looking at “epi” and “non epi” forms, the retention order is reversed between the free and the esterified compounds, the epi forms elute after for CG and C, whereas they elute before for GCG and CG. Depending on the specificity of the stationary phase, the retention order between EGCG and EC can change. Moreover, despite the satisfactory separation of standards, many coelutions could occur when working on real extracts [6,15,16,17].

Because the catechin content is associated with the quality of tea [19], many studies were conducted on the extracts obtained with hot water maceration, or with various modern extraction methods to study the catechin content of green and black teas, as well as how these techniques degrade these substances [1,6,7,8,9,10,11,12,13,14,15,16,18,20,21,22,23,24]. The methods are microwave-assisted extraction (MAE) [7,17,24], ultrasound-assisted extraction (UAE) in water or ethanol/ethyl acetate [12,21,23], liquid–solid extraction with varied solvents (methanol and ethanol) and/or mechanical help [11,13,18,23], ultra-high-pressure-assisted solvent extraction (UPSE) (10 to 50 MPa) [19,25], pressurized-liquid extraction (PLE) with methanol [21] or ethyl lactate/water mixtures [26], and last but not least, supercritical fluid extraction (SFE) [2,3,4,5,16,20,27,28,29,30,31] with CO_2_-based fluids.

In addition, caffeine found in *Camellia sinensis* leaves may have an adverse effect on health [2,3,4,5]. Therefore, caffeine is often removed from the tea leaves, mainly by using SFE [2,3,4,5,16,27,28,29,30], or with solid-phase adsorption of the liquid extracts [24,32]. However, the decaffeination process can co-extract catechins from the plant, thereby reducing the positive effect of the consumed beverage. The application of SFE on green tea compounds has previously been studied and results showed that the highest recovery yield for both caffeine and catechins was obtained with the ethanol:CO_2_ (5:95; *v:v*) composition. This was combined with a temperature of 80 °C and a pressure of 30 MPa [3]. However, the highest selectivity was achieved at 63 °C and 23 MPa, with an extraction yield of 96% for caffeine and 40.6% for catechins [28]. In another study, the effects of particle size, temperature, modifier (EtOH) volume, and extraction duration were examined through an orthogonal array design of nine experiments. The results reported that the particle size of 0.2–0.6 mm allowed the recovery of 70% of caffeine and only 6% of catechins at 80 °C, 30 MPa, and a CO_2_ flow rate of 1.5 mL/min for 2 h, using a total ethanol volume of 30 mL [27]. An ANOVA study of the results, based on the nine experiments, showed that temperature, particle size, extraction time, and modifier volume had a significant influence on caffeine removal, but none of these parameters affect the concentration of EGCG, the main catechin. In addition, the smallest particle size (0.23 mm) favored the removal of caffeine, whereas the largest particle size (5.5 to 17.4 mm) reduced the extraction of EGCG [3].

The use of water as a modifier degraded selectivity, yielding 70–80% recovery for the caffeine extraction and 60–70% for catechins [2,4,27]. According to a different study, the dried leaves swelled up when water was added to CO_2_, enhancing the solute diffusion and increasing the recovery of caffeine and catechin extraction. Additionally, the extraction recovery increased with the temperature, the pressure, and the water content, whereas the extraction selectivity increased with pressure and water content but decreased with the increase in temperature [5].

All these findings demonstrated that the addition of ethanol or water to SC-CO_2_ is necessary to significantly enhance the solubility of polar compounds, caffeine, and/or catechins [2,4,5,20,27,32]. When considering the kinetics of extraction using ethanol as a modifier, no variations in selectivity can be noted between 30 min and 3 h [3,27,28]. Moreover, the extraction kinetics of catechins, caffeine, and chlorophylls were very similar, regarding pressure, temperature, and modifier concentration [28].

Recently, ethyl lactate, ethyl acetate, and ethanol used as modifiers were compared at 30 MPa and 70 °C [32]. Ethyl lactate displayed the highest extraction recovery for caffeine, while ethyl acetate seemed better suited to achieve a higher selectivity of caffeine vs. catechins [31].

This paper describes the use of a rationalized approach to ensure the best recovery of polar compounds from green tea leaves, including caffeine and catechins, by reducing the amount of used solvent with classical maceration extraction to provide extracts for cosmetic uses. Considering the advantages of SC-CO_2_, including the provision of concentrated extracts after the CO_2_ depressurization during sample collection and its ecological nature, SFE with ethanol (EtOH) and EtOH:H_2_O modifiers were chosen to achieve this goal. From previous studies conducted in our laboratory, either on the extraction of flavonoids from heartwood [33] or on the selective extraction of bioactive compounds from rosemary [34], extraction temperature, modifier percentage, and percentage of water in ethanol as additive and modifier were chosen as optimization parameters.

## 2. Results and Discussion

SFE on tea leaves was optimized based on a Box–Behnken design of the experiment (BBD). The percentages of modifier and water as an additive were selected because of their capacity to change the polarity of the extraction phase, while the temperature was chosen for its ability to influence the solubility of compounds in the SC-CO_2_ (Table 1).

In our previous works, a BBD was employed to optimize the extraction of two polar flavonoids, where pressure was an optimization parameter ranging from 10 to 20 MPa [33]. It showed that pressure was an insignificant variable in that range for the extraction of such polar compounds. This was mainly due to the high density of the fluid with the addition of high percentages of modifier (up to 30%). As it showed little influence on the model’s response (compound yield), pressure was kept constant at 15 MPa during the present study and was not investigated.

### 2.1. Statistical Analysis, Model, and Factor Significance

The quadratic model’s determination coefficient (R^2^) was equal to 0.89, while the value of adjusted determination coefficient (adj R^2^) was 0.82. This signifies that the model was unable to account for 11% of the total variance. The R^2^ value demonstrates a strong correlation between the expected and experimental response values and indicates that the empirical model shows a good fit with experimental data. The relative standard deviation (RSD %) of the central point replicates (five repetitions) for the total molecules extracted from green tea leaves was equal to 6.68 %. Furthermore, the Dixon statistical test was applied and showed no outliers. This was also confirmed with the residual analysis. The correlation between the three variables (X_1_, X_2_, and X_3_) and the total yield of the molecules of interest (Y) (Table 1) is presented in Equation (1).

Y (mg/g) = −162.8 + 3.187X_1_ + 6.547X_2_ + 6.453X_3_ − 0.02537X_1_^2^ − 0.112X_2_^2^ − 0.1673X_3_^2^ (1)

The summary of the analysis of variance (ANOVA) for the polynomial model is shown in Table 2 and the regression values showed that the model is significant. The probability related to F-value and *p*-value were used to determine the significance of each factor, and their contribution percentages were studied to determine their impact on the model’s response. The total yield of molecules of interest from the *Camellia sinensis* regression model had an F-value of 13.55 with a related *p*-value of 0.0003. Both statistical terms indicate a significant model.

The factors X_2_ (modifier percentage) and X_3_ (H_2_O%) showed the highest significance, with a contribution of 12% and 42%, respectively. This was also validated by their *p*-values of 0.0385 and 0.0010, respectively. This indicates that even though the modifier percentage is significant, the contribution of water percentage to the total yield was higher. Moreover, X_1_ (temperature) had a low significance on the total yield, with a 5% contribution to the model and a *p*-value of 0.125. This suggests that the temperature variation had no substantial impact on the yield of caffeine and total catechins.

### 2.2. Effects of the Extraction Parameters Assessed with BBD

Heat maps were used as a graphical representation of the independent factors and dependent response interaction and correlated to the response of green tea compound yield (mg/g) for 45 min of extraction time.

**Figure 2:**
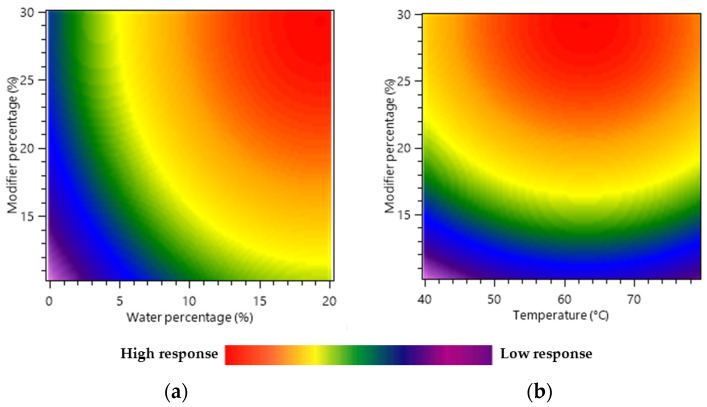
Response surface heat maps showing the interactive effects of the variables on the total molecule yield (mg/g) of green tea leaf extracts. (**a**) Interaction between the modifier’s percentage (X_2_) and water in modifier percentage (X_3_), (**b**) interaction between the modifier’s percentage (X_2_) and temperature (X_1_).

Figure 2a illustrates the effect of the modifier’s percentage and water content on the extract’s yield. This revealed a positive impact of both the modifier’s percentage and the water content on the response. When the modifier percentage increased from 10% to 30%, the total molecule yield increased from 39.15 mg/g to 80.48 mg/g when the temperature was set at 60 °C and 10% of H_2_O was included in the modifier. This represented an increase of 105% in the yield. Moreover, as the H_2_O % varied from 0 to 20%, the predicted total yield varied from 23.22 to 85.34 mg/g. These results confirmed the significant effect of water as an additive, as the increase in this case was 267% compared with the 105% with the modifier’s percentage.

This behavior is probably due to the high polarity of caffeine and catechins. Indeed, the low polarity of SC-CO_2_ is limiting its extraction capability to non-polar or moderately polar molecules. However, the addition of ethanol and water as modifiers increased the polarity of the extraction phase and, consequently, its affinity for polar compounds like the polyphenols of interest, therefore increasing the recovery yield.

Temperature is generally an essential factor in SFE optimization. It can affect the solubility of polar compounds in SC-CO_2_, the diffusion rate of the compounds, and the properties of the solvent (density and viscosity). Generally, higher temperatures increase the solubility and in some cases the velocity of polar compound diffusion from the plant matrix into the extraction phase leading to higher extraction yields [35]. However, at excessively high temperatures, the polar compounds may degrade, affecting the quality of the extracted compounds. Moreover, increasing the temperature can also decrease the density of the SC-CO_2_, reducing its ability to penetrate the tea matrix and solubilize the target compounds [36]. Consequently, Figure 2b demonstrated that the effect of temperature on tea compound recovery was insignificant, in accordance with ANOVA. When working with modifier percentages from 10 to 30%, with or without water, the fluid compressibility is low, meaning that changes in temperature cannot induce large fluid density variation or solubility changes.

### 2.3. Extraction Optimum of Caffeine and Catechins

Based on Equation (1), the computed optimum parameters offering maximal yield of caffeine and total catechins are as follows: temperature of 62 °C, a modifier percentage of 29.3%, and a water percentage in the modifier of 19.3%.

These conditions are close to the extraction parameters of experiment n°12, which allowed the recovery of 90.1 mg/g of green tea compounds of interest from the biomass. The extraction kinetics (Table 1) of this experimental point showed that most green tea target compounds were extracted within 15 min. This indicated that, in these conditions, 15 min is sufficient, from an economical point of view, for the extraction of most of the molecules of interest. Additionally, other experimental points offered a high extraction yield of the target compounds. For instance, experiments n°4 (80 °C, 30% EtOH:H_2_O 90:10) and n°7 (40 °C, 20% EtOH:H_2_O 80:20) had yields of 92.73 and 87.19 mg/g, respectively. These conditions could be selected with a goal to reduce both the extraction temperature (energetically favorable) and the modifier consumption. However, both experiments offered slower extraction kinetics compared with experiment n°12, where in 15 min the recovery yields were 80.3%, 67%, and 94% for experiments n°4, n°7, and n°12, respectively. These recovery differences towards the extraction duration are related to different kinetics. For experiment n°4, the use of only 10% of water included in the modifier could reduce the solubility of the compounds, as was also observed in experiments 13 to 17 (central point of the BBD). On the contrary, the lower temperature for experiment n°7 mainly changed the diffusion inside the matrix, as was also observed for experiment n°3.

### 2.4. Comparison between Supercritical Fluid Extraction (SFE) and Ultrasound-Assisted Extraction (UAE)

The SFE method was compared with UAE, which is considered an efficient and simple extraction technique. This comparison was conducted in equivalent conditions: 13.5 mL of solvent volume (equivalent to 30% of modifier for 15 min at 3 mL/min flow) and 15 min of extraction time.

**Figure 3:**
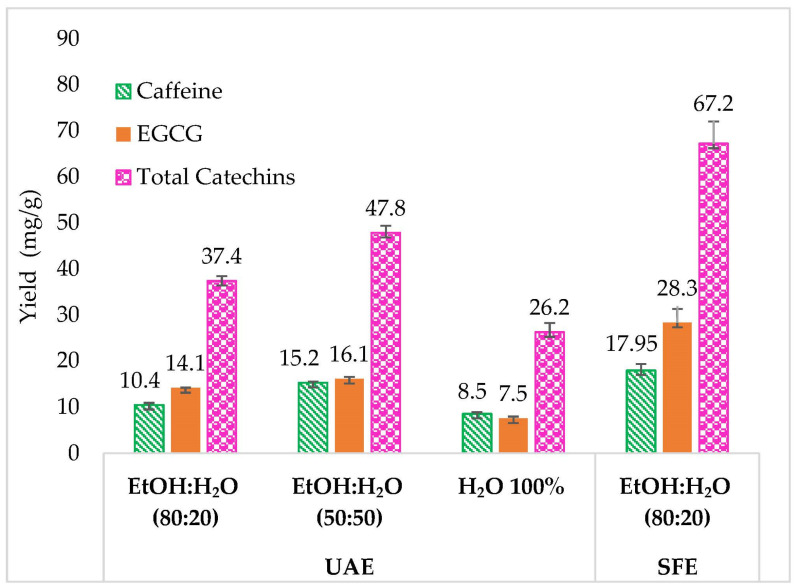
Comparison between supercritical fluid extraction (SFE) and ultrasound-assisted extraction (UAE) for green tea leaf compounds (total catechins represents all the catechins including EGCG).

Three EtOH:H_2_O ratios were explored: 80:20, 50:50, and 0:100 (or 100% H_2_O). The first conditions were chosen to compare the extraction yields in similar ratios of EtOH: H_2_O for the optimized SFE conditions (EtOH:H_2_O 80:20), and the second from conditions classically observed in the literature. The third composition (100% H_2_O) was selected to investigate the effect of higher H_2_O% on the yield of green tea target compounds. The results are presented in Figure 3. They demonstrate that both extraction methods can extract green tea compounds. However, different yields were obtained from both techniques.

Using EtOH:H_2_O 80:20 *v:v*, SFE provided higher extraction yields for Caff, EGCG, and total catechins with 17.95, 28.34, and 67.23 mg/g, respectively, compared with 10.44, 14.11, and 37.39 mg/g with UAE.

The UAE results showed that an increase of 30% of water (between EtOH:H_2_O 80:20 and 50:50 *v:v*) had a positive effect on the extraction yield of the target compounds. The percentage of Caff, EGCG, and total catechins increased by 46%, 14.18%, and 27.8%, respectively. However, the UAE with 100% water had an opposite effect on the extraction yield of green tea compounds, as decreases of 44.07%, 53.41%, and 45.18% for caffeine, EGCG, and total catechin extraction, respectively, was observed, compared with EtOH:H_2_O 50:50 *v:v*.

Hence, for the same liquid solvent consumption and composition (EtOH:H_2_O 80:20), the use of SFE increased the extraction yield by 72.11%, 100.7%, and 79.68% for Caff, EGCG, and total catechins, respectively. When comparing the two best extraction recoveries, with 50:50 EtOH:H_2_O for UAE and 80:20 EtOH:H_2_O for SFE, the higher amount of compounds was still obtained via SFE. Consequently, in the goal to produce ingredients for cosmetic products containing both caffeine and catechins, SFE can advantageously substitute UAE, leading to more concentrated extracts. Furthermore, SFE affords a lower water content (20% toward 50%). Since water requires more energy to evaporate, SFE extracts can also provide a reduction in energy consumption for the final evaporation step, and this lower water content also favors compound stability.

### 2.5. Selective Extraction of Caffeine

The selective extraction of caffeine from green tea or other caffeine-rich plants was previously reported in other studies [2,3,4,5]. However, for these studies the core focus was the decaffeination of the biomass while avoiding the extraction of catechins, to provide extracts containing only the bioactive catechins. Nevertheless, as previously reported in the introduction, regardless of the experimental conditions, the catechin concentration was dramatically reduced after the full extraction of caffeine.

In the present study, the results of the experimental design (Table 1) showed that the conditions of the experiments n°1 (40 °C, 10% EtOH:H_2_O 90:10), 6 (80 °C, 20% EtOH) and 9 (60 °C, 10% EtOH), offered a selective extraction of caffeine.

Therefore, a selective extraction of caffeine was explored followed by an additional step to extract the remaining (minor amounts of) caffeine and the total catechins. Since we had previously selected experiment n°12 (60 °C EtOH:H_2_O 80:20) as the best suited for the overall extraction of the total compounds, this condition was chosen for the second step of the sequential extraction. Therefore, we retained experiment n°9, with a temperature of 60 °C, for the extraction of caffeine in the first step, to keep a constant temperature during the sequential extraction.

**Figure 4:**
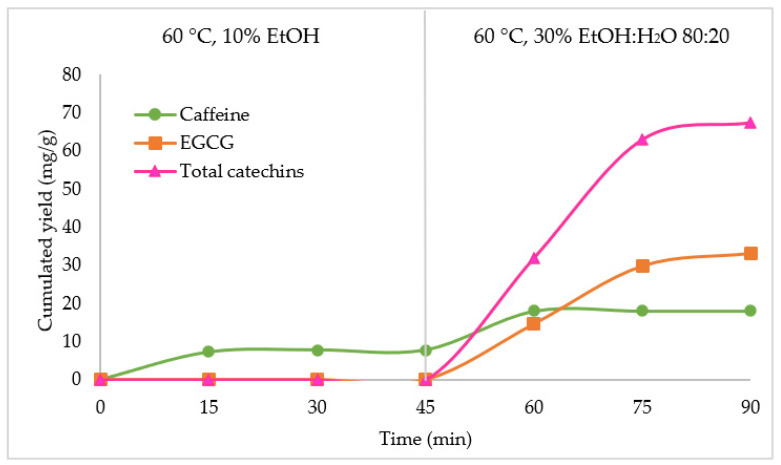
SFE sequential selective extraction of caffeine from green tea leaves (total catechins represents all the catechins including EGCG).

Then, the sequential selective extraction kinetics was applied to 1 g of green tea biomass, with the first step using 60 °C and 10% EtOH as a modifier for 45 min (left part of Figure 4). This first step allowed the recovery of 44.55% of the green tea leaf extractible caffeine in 15 min; this was consistent with the amount extracted in experiment n°9. This means that the first step was able to recover almost half of the total caffeine content. The recovery of caffeine did not increase when the extraction time was extended from 15 to 45 min.

A second step was applied using the optimized SFE extraction conditions for the target compounds, with 60 °C and 30% EtOH:H_2_O 80:20 for 45 min, corresponding to the conditions of experiment n°12, the one displaying the highest recovery for all the compounds and catechins in the first 15 min of extraction. In the right part of Figure 4, we can observe that an additional amount of caffeine was extracted, leading to a final yield of 18.02 mg/g, which was approximatively the same value as the one reached for the best caffeine extraction (Table 1). This second step also resulted in the extraction of catechins with a final yield of 67.54 mg/g. For these compounds, it was observed that the application of a first selective step to extract caffeine slowed and reduced the catechin extraction kinetics in the second step. Figure 4 showed that in the first fraction of the second extraction step only 32.04 mg/g of catechins were extracted. In addition, the second fraction of 15 min (30 min total extraction time) allowed to recover twice as much with 63.12 mg/g cumulated yield. However, in the experimental design, with experiment n°12 only 15 min were needed to extract the major part of catechins (Table 1). Furthermore, after 45 min, the total amount of extracted catechins was 67.54 mg/g, whereas it reached 72.15 mg/g for experiment n°12.

This seems to indicate that the first step with pure ethanol as the modifier could induce a reduction in the compound accessibility into the matrix. According to other research, adding water as a modifier caused the matrix to swell, which encouraged the diffusion of the extraction solvent and, consequently, the release of substances through the swollen channels of the solid matrix [2,5]. As a result, when starting with pure ethanol as a cosolvent, the effectiveness of the second step of sequential extraction is decreased since the first step’s absence of water possibly prevented the matrix from swelling.

As a conclusion, from the three-parameter experimental design, a sequential selective extraction was conducted and two fractions were obtained, with the first one containing only caffeine and the second one containing the rest of the caffeine and around 80% of the total catechin content.

## 3. Materials and Methods

### 3.1. Plant Material

The green tea plant material (*Camellia sinensis* (L.) Kuntze) consisted of the dried leaves milled into a powder of a dark green color. The plant material was supplied by PMA 28 (Varize, France) and stored at room temperature in an airtight container.

### 3.2. Chemicals and Reagents

CO_2_ gas was supplied by Air Liquide (Fleury-les-Aubrais, France). Acetonitrile (ACN) and methanol (MeOH) were used for the mobile phase. Ethanol (EtOH) was used as the extraction solvent and sample diluent. All solvents were supplied by VWR (Fontenay-sous-Bois, France). Formic acid (FA) added to the mobile phase was obtained from Sigma-Aldrich (Merck, Semoy, France). Ultra-pure water (H_2_O) was purified with a Milli-Q system from Sigma-Aldrich, St. Louis, MO, USA, with a resistance higher than 18 MW. Ethylenediaminetetraacetic acid (EDTA) and ascorbic acid (AA) used as stabilizing agents were supplied by Sigma-Aldrich.

The standards used to identify and/or quantify green tea target compounds (Figure 1) such as (−)-Epicatechin gallate (ECG), (−) Epigallocatechin (EGC), and (−)-Epicatechin (EC) were supplied by Extrasynthese (Genay, France). (−)-Epigallocatechin gallate (EGCG), (+)-Gallocatechin (GC), and (+)-Catechin (Cat) were acquired from Sigma-Aldrich. And finally, caffeine (Caff) was supplied by Thermo-Fisher Scientific (Artenay, France).

### 3.3. SFE Extraction

All extractions were performed using a Waters MV-10 ASFE. In the stainless steel extraction vessel (5 mL), 1 g of plant material was combined with 1 g of diatomaceous earth powder from Sigma-Aldrich (Merck, France). Cotton was added to the top and bottom of the cell: its role is to filter the extract and to fill the cell completely. A static extraction time of 2 min was applied at the beginning of each experiment, it was then followed by continuous dynamic extraction, with a flow of 3 mL/min. The amount of modifier added was determined based on the overall extraction flow.

For each experiment in the BBD, three fractions of 15 min were collected, with a total extraction duration of 45 min. The concentrations for each fraction are shown in Table 1. The pressure was kept constant at 15 MPa.

Table 1 presents the list of the experiments with the responses represented by the yields (mg/g) of the major green tea compounds separately, Caff and EGCG; total catechins represents all the catechins mentioned in Figure 1 including EGCG. The total molecule yield is the sum of total catechins and the caffeine yield. This terminology is applied to all the results in this study.

### 3.4. Ultra-Sound-Assisted Extraction (UAE)

Using 1 g of powdered dried green tea leaves, the UAE was carried out in a Branson 3510 (Bransonic^®^ ultrasonic, Semoy, France) bath from Sigma-Aldrich (Merck, France). The power of the UAE was equal to 130 W. The extraction solvent used was a combination of EtOH:H_2_O in various ratios, including 80:20, 50:50, and 0:100 *v:v*. The plant and 13.5 mL of solvent (see Section 3.4) were combined and then sonicated at room temperature for 15 min. The extracts were centrifuged at 10,000 rpm for 10 min at 25 °C and filtered using a 0.45 µm PTFE syringe filter from Agilent Technologies (Les Ulis, France). For UHPLC analysis, they were subsequently diluted four times with the stabilization solvent.

### 3.5. UHPLC-UV Analysis

All extracts were analyzed with a Nexera-LC40 system of Shimadzu Corporation (Kyoto, Japan). This system was equipped with a photodiode array (PDA) detector (SPD-40), a solvent delivery unit (LC-40), an auto-sampler (SIL-40), a column oven (CTO-40), and a system controller SCL-40. All chromatograms were recorded on LabSolutions LC-UV 5.97 SP1 version (Shimadzu Corporation, Kyoto, Japan).

A Cortecs C18 (100 × 3.0 mm) column coupled to a Cortecs C18 VanGuard Cartridge (5 × 3.0 mm), both packed with 2.7 µm superficially porous particles from Waters Corporation (Milford, MA, USA), were used for the analysis of all extracts. The column temperature was maintained at 30 °C. The injection volume was 5 µL. The flow rate was maintained at 1 mL/min. The equilibration time between two injections was 5 min.

#### 3.5.1. Elution Gradient and Quantification

The total time of each analysis was 18 min; the mobile phase consisted of a combination of H_2_O (solvent A) and MeOH (solvent B), both acidified with 0.1% of FA and ACN (solvent C).

**Figure 5:**
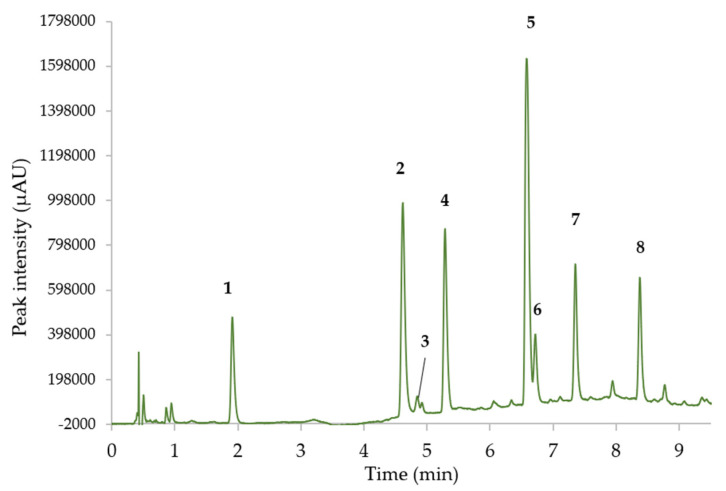
UHPLC separation of green tea extract (210 nm). Peak identifications: (1) GC (Tr = 1.91 min), (2) EGC (Tr = 4.61 min), (3) Cat (Tr = 4.84 min), (4) Caff (Tr = 5.29 min), (5) EGCG (Tr = 6.58 min), (6) EC (Tr = 6.72 min), (7) GCG (Tr = 7.35 min), and (8) ECG (Tr = 8.38 min).

Solvent C percentage was kept constant at 3% during the whole analysis, while the percentage of solvent B varied as follows: 0–2 min: 0% B, 2–9 min: 0–30% B, 9–12 min: 87% B, and 12–18 min 0% B. The separation of the target molecules and their retention time (Tr min) is presented in Figure 5. Detection and quantification were conducted at 210 nm, which allowed for the identification of all catechins, especially GCG and ECG. In addition, the gallic acid standard was eluted using this method (Tr = 0.83 min); however it was not detected in SFE extracts analyzed during this study.

The identification of peaks 2, 3, 4, 5, 6, and 8 was conducted using the means of standards; as for peaks 1 and 7 it was estimated according to the compound’s retention behavior in liquid chromatography according to the literature [6,7,8,9,10,11,12,13,14,15,16,17,18]. As usually found in previous papers, the retention order of catechins followed the classical pattern GC/EGC/C/EGCG/EC/GCG/ECG. As expected, the main compounds were the epi forms, EGC (peak 2), and EGCG (peak 5), and despite the high UV absorbance of many compounds at 210 nm, some coelutions of matrix peaks were observed.

Quantitative analyses of caffeine (Caff) were performed by injecting a standard at 11 different concentrations from 0.001 mg/mL to 0.5 mg/mL. The calibration curve was obtained at 210 nm (y = 3 × 10^7^x + 378,990, R^2^ = 0.9917) and the equation was used to estimate the concentrations from the peak areas of Caff (y = concentration; x = peak area).

Quantification of EGCG and the total catechins was obtained in the same manner. It was performed by injecting a standard at 9 different concentrations from 0.001 mg/mL to 0.2 mg/mL. The calibration curves were obtained at 210 nm. The EGCG equation (y = 4 × 10^7^x + 188,476, R^2^ = 0.9885) was used to estimate the concentrations from the peak areas of EGCG. As for the other catechins, the concentrations were quantified as an equivalent to EGCG. This approximation was possible due to their structural similarities and the similar molar absorption coefficient (ε) at 210 nm, which was studied from the spectra of standards provided by the diode array detector.

#### 3.5.2. Sample Treatment

To compare and determine the extraction yields for the target compounds, all extracts were diluted in volumetric flasks of 10 mL or 20 mL with ethanol to have a controlled dilution volume. The extracts were then filtered using a 0.45 µm PTFE syringe filter from Agilent Technologies (Les Ulis, France) and diluted 4 times in the stabilization solvent consisting of H_2_O:ACN 9:1 *v:v* with EDTA and AA at a concentration of 0.25 mg/mL each. This stabilization solvent was used for several reasons: to avoid any degradation of the molecules (EDTA) [37], to homogenize and avoid any precipitation of the extracts during the analysis (AA) [38], and to adapt the injection solvent to the beginning of the gradient elution (ACN). This was also validated by a study of stability conducted in our laboratory, where the concentration of extracts was assessed during 12 days at 5 °C with pure water or with the stabilization solvent. It showed that the extracts diluted in water degraded by 50% and 12% for EGCG and catechins, respectively. However, with the stabilization solvent, the degradation after 12 days in the fridge was only 10% and 6% for EGCG and catechins, respectively. Caffeine showed minimal degradation in both solvents.

The peak areas and concentrations were later normalized for all data treatments (Equation (2)) to the concentrations of the target compounds per gram of biomass used (Y).

Y (mg/g) = concentration of diluted extracts (mg/mL) × dilution flask volume (mL) × 4 (2)

### 3.6. Experimental Design and Statistical Analysis

Ellistat software 6.4 2020/11 version (Annecy, Auvergne-Rhône-Alpes) was used for the experimental design and data analysis. A Box–Behnken design (BBD) with response surface methodology (RSM) was chosen to establish the model and determine the response pattern. The BBD was used to optimize the supercritical fluid extraction of target compounds from green tea leaves. The three independent factors used in this study were temperature (X_1_), total modifier percentage (X_2_), and water percentage added as an additive to the modifier (X_3_), with three levels coded (+1) for the highest, (0) for the middle, and (−1) for the lowest levels.

The same software was employed for the graphical analysis of the data and the regression model. The analysis of the variance using ANOVA was used to determine the significance of the independent variables on the response. This was performed using statistical values like Fisher’s test (F value) and significant factors were defined by a *p*-value < 0.05. Correlation coefficient (R^2^) and adjusted correlation coefficient (adj R^2^) were employed to assess the fitness of the design model. To visualize the interaction between the variables, heatmap plots were utilized. Optimal extraction conditions were determined by solving the regression equation that represented the predictive model.

Flow rate and plant mass were kept constant in all SFE experiments. The dependent response or output (Y) was the sum of the yield of all target compounds (mg/g) for all three fractions. The experiments were randomized to maximize the effect of the variability in the response. Five replicates at the middle experimental conditions (X_1_ = 0, X_2_ = 0, and X_3_ = 0) of the design were conducted to evaluate the experimental repeatability. The standard deviation (SD%) was estimated to confirm the reproducibility of the extraction model; therefore, the data were represented as mean values ± standard deviation.

The polynomial Equation (3) represents the relationship between the response and the three independent variables (X_1_, X_2_, and X_3_).

Y (mg/g) = β_0_ + β_1_ X_1_ + β_2_ X_2_ + β_3_ X_2_ + β_11_ X_1_^2^ + β_22_ X_2_^2^ + β_33_ X_3_^2^ (3)

β_11_, β_22_, and β_33_ = quadratic coefficients and X_1_, X_2_, and X_3_ represent the factors temperature, modifier percentage, and water percentage in the modifier, respectively.

## 4. Conclusions

Overall, the supercritical extraction of polar compounds from green tea leaves (caffeine and polyphenols) was optimized by means of the design of a series of experiments. It validated the benefits of SC-CO_2_ mixed with polar solvents, mainly EtOH:H_2_O for the high-yield isolation of extracts rich in polar compounds. Our results demonstrated that modifier percentage and especially water percentage are the most influential parameters to increase the total yield of the target molecules of green tea, while temperature had no significant effect. The optimized conditions allowed for the recovery of 90.10 mg/g of the compounds of interest.

The comparison between SFE and UAE showed that modified SC-CO_2_ extraction is an effective extraction technique for the recovery of green tea leaf target compounds that could lead to a reduction in the total solvent consumption from an industrial point of view when working with a greater amount of plant material. Our results show that for the same composition of organic solvent used with both extraction methods (EtOH:H_2_O, 80:20, *v*:*v*), SFE presented increases in the extraction yields in the amounts of 72.11, 100.7, and 79.68% for caffeine, EGCG, and total catechins, respectively.

A selective extraction of caffeine was also achieved, which allowed to recover 40.55% of extractible caffeine in the first step. However, a small loss of 5.37% in total catechin recovery was observed after the decaffeination step, compared with the extracted amount obtained directly from the optimal extraction conditions (experiment n°12); furthermore, an increase in the extraction duration, from 15 to 45 min, was observed for this second step.

In conclusion, SFE allowed for the recovery of high yield extracts with a reduced organic solvent consumption and a partial selective extraction of caffeine. Thus, the proposed approach can be applied not only to provide a caffeine-rich extract but additionally a catechin-rich one, both for various cosmetic and health uses.

## Figures and Tables

**Table 1:** Design matrix in the Box–Behnken model and responses (represented with SD values for each response) for green tea extracts (total catechins represents all the catechins including EGCG).

Exp N°	Coded Levels			Results (Concentration mg/g of Biomass after 45 min of Extraction)									Total Molecules (mg/g of Biomass)		
	X_1_	X_2_	X_3_	Caff	GC	EGC	Cat	EGCG	EC	ECG	Total Catechins	Total Molecules (Y mg/g)	Fraction 1 (15 min)	Fraction 2 (30 min)	Fraction 3 (45 min)
	Temperature (°C)	Modifier (%)	H_2_O in Modifier (%)												
1	40 (−1)	10 (−1)	10 (0)	13.04 ± 1.01	0.18 ± 0.01	0.09 ± 0.01	0.04 ± 0.00	0.12 ± 0.01	0.31 ± 0.02	0.12 ± 0.01	0.86 ± 0.06	**13.90 ± 0.93**	8.94 ± 1.19	3.68 ± 0.66	1.28 ± 0.20
2	80 (+1)	10 (−1)	10 (0)	13.13 ± 1.01	0.46 ± 0.02	2.46 ± 0.13	0.14 ± 0.01	4.53 ± 0.43	1.34 ± 0.08	1.88 ± 0.11	10.80 ± 0.72	**23.93 ± 1.60**	9.16 ± 1.22	3.42 ± 0.62	11.36 ± 1.75
3	40 (−1)	30 (+1)	10 (0)	17.24 ± 1.33	1.50 ± 0.05	14.40 ± 0.76	0.55 ± 0.03	22.75 ± 2.18	5.27 ± 0.30	6.39 ± 0.38	50.87 ± 3.40	**68.11 ± 4.55**	42.63 ± 5.69	15.52 ± 2.80	9.96 ± 1.53
4	80 (+1)	30 (+1)	10 (0)	19.75 ± 1.53	2.14 ± 0.08	20.35 ± 1.07	0.78 ± 0.04	32.84 ± 3.14	7.08 ± 0.40	9.79 ± 0.58	72.98 ± 4.88	**92.73 ± 6.20**	74.51 ± 9.94	12.14 ± 2.19	6.08 ± 0.93
5	40 (−1)	20 (0)	0 (−1)	3.59 ± 0.28	0.11 ± 0.00	0.94 ± 0.05	0.05 ± 0.00	1.17 ± 0.11	0.54 ± 0.03	0.57 ± 0.03	3.38 ± 0.23	**6.97 ± 0.47**	4.93 ± 0.66	1.15 ± 0.21	0.89 ± 0.14
6	80 (+1)	20 (0)	0 (−1)	9.42 ± 0.73	0.22 ± 0.01	1.55 ± 0.08	0.12 ± 0.01	2.07 ± 0.20	1.14 ± 0.07	0.99 ± 0.06	6.10 ± 0.41	**15.52 ± 1.04**	9.60 ± 1.28	3.29 ± 0.59	2.63 ± 0.40
7	40 (−1)	20 (0)	20 (+1)	16.78 ± 1.30	1.99 ± 0.07	19.13 ± 1.01	1.19 ± 0.05	32.07 ± 3.07	6.46 ± 0.37	9.57 ± 0.56	70.41 ± 4.70	**87.19 ± 5.83**	58.42 ± 7.79	23.67 ± 4.27	5.10 ± 0.78
8	80 (+1)	20 (0)	20 (+1)	13.56 ± 1.05	2.12 ± 0.08	14.56 ± 0.77	1.40 ± 0.06	20.72 ± 1.98	6.27 ± 0.36	8.23 ± 0.48	53.30 ± 3.56	**66.86 ± 4.47**	55.97 ± 7.47	9.20 ± 1.66	1.70 ± 0.26
9	60 (0)	10 (−1)	0 (−1)	10.45 ± 0.81	0.03 ± 0.00	0.00 ± 0.00	0.00 ± 0.00	0.00 ± 0.00	0.00 ± 0.00	0.00 ± 0.00	0.03 ± 0.00	**10.49 ± 0.70**	8.80 ± 1.17	1.05 ± 0.19	0.64 ± 0.10
10	60 (0)	30 (+1)	0 (−1)	6.37 ± 0.49	0.34 ± 0.01	3.29 ± 0.17	0.15 ± 0.01	4.30 ± 0.41	1.34 ± 0.08	1.43 ± 0.08	10.85 ± 0.72	**17.22 ± 1.15**	10.44 ± 1.39	4.07 ± 0.73	2.71 ± 0.42
11	60 (0)	10 (−1)	20 (+1)	13.77 ± 1.06	1.58 ± 0.06	11.62 ± 0.61	1.06 ± 0.05	15.33 ± 1.47	4.71 ± 0.27	6.44 ± 0.38	40.74 ± 2.72	**54.51 ± 3.64**	12.90 ± 1.72	26.67 ± 4.81	14.94 ± 2.30
12	60 (0)	30 (+1)	20 (+1)	17.95 ± 1.39	2.00 ± 0.07	20.44 ± 1.08	1.33 ± 0.06	31.55 ± 3.02	6.88 ± 0.39	9.95 ± 0.59	72.15 ± 4.82	**90.10 ± 6.02**	85.10 ± 11.35	3.90 ± 0.70	1.11 ± 0.17
13	60 (0)	20 (0)	10 (0)	16.02 ± 1.24	1.66 ± 0.06	14.93 ± 0.79	0.63 ± 0.03	20.70 ± 1.98	5.68 ± 0.32	7.28 ± 0.43	50.89 ± 3.40	**66.91 ± 4.47**	45.64 ± 6.09	12.04 ± 2.17	9.23 ± 1.42
14	60 (0)	20 (0)	10 (0)	16.91 ± 1.31	1.76 ± 0.06	15.47 ± 0.82	0.68 ± 0.03	20.87 ± 2.00	6.49 ± 0.37	8.04 ± 0.47	53.32 ± 3.56	**70.23 ± 4.69**	43.18 ± 5.76	14.63 ± 2.64	12.42 ± 1.91
15	60 (0)	20 (0)	10 (0)	17.74 ± 1.37	1.75 ± 0.06	15.33 ± 0.81	0.66 ± 0.03	20.64 ± 1.98	6.29 ± 0.36	7.89 ± 0.46	52.55 ± 3.51	**70.29 ± 4.70**	45.53 ± 6.07	14.66 ± 2.64	10.11 ± 1.55
16	60 (0)	20 (0)	10 (0)	19.70 ± 1.52	1.83 ± 0.07	16.76 ± 0.88	0.70 ± 0.03	25.01 ± 2.39	6.58 ± 0.38	8.56 ± 0.50	59.43 ± 3.97	**79.13 ± 5.29**	55.41 ± 7.39	15.46 ± 2.79	8.26 ± 1.27
17	60 (0)	20 (0)	10 (0)	17.85 ± 1.38	1.72 ± 0.06	14.63 ± 0.77	0.63 ± 0.03	19.83 ± 1.90	6.12 ± 0.35	7.74 ± 0.46	50.67 ± 3.39	**68.52 ± 4.58**	38.77 ± 5.17	19.64 ± 3.54	10.11 ± 1.55

**Table 2:** ANOVA for response surface regression model for total yield of target molecules (mg/g) from green tea leaf extracts.

Source	DF	SS	MS	F Value	Prob > F	Contribution (%)	Conclusion
Regression model	6	13,574	2262.3	13.5449	0.0003		Very significant
X_1_-Temperature (°C)	1	65.38	468.32	2.804	0.125	5	Not significant
X_2_-Modifier (%)	1	3416.8	1092.2	6.5391	0.0285	12	**Significant**
X_3_-H_2_O (%)	1	7716.5	3873.1	23.1893	0.0007	42	**Very significant**
X_1_^2^	1	579.65	433.5	2.5955	0.1382	5	Not significant
X_2_^2^	1	616.39	528.33	3.1633	0.1057	6	Not significant
X_3_^2^	1	1179.1	1179.1	7.0595	0.024	13	**Significant**
Residuals	10	1670.2	167.02			18	
Total	16	15,244					
	Degrees of freedom	Sum of squares	Mean square				

## Data Availability

Data will be available upon request.
